# The Lungs in Space: A Review of Current Knowledge and Methodologies

**DOI:** 10.3390/cells13131154

**Published:** 2024-07-06

**Authors:** Michaela B. Smith, Hui Chen, Brian G. G. Oliver

**Affiliations:** 1 Respiratory Cell and Molecular Biology Group, Woolcock Institute of Medical Research, Macquarie Park, NSW 2113, Australia; michaela.smith@woolcock.org.au; 2 School of Life Science, University of Technology Sydney, Ultimo, NSW 2007, Australia; hui.chen-1@uts.edu.au

**Keywords:** microgravity, respiratory, lungs, space

## Abstract

Space travel presents multiple risks to astronauts such as launch, radiation, spacewalks or extravehicular activities, and microgravity. The lungs are composed of a combination of air, blood, and tissue, making it a complex organ system with interactions between the external and internal environment. Gravity strongly influences the structure of the lung which results in heterogeneity of ventilation and perfusion that becomes uniform in microgravity as shown during parabolic flights, Spacelab, and Skylab experiments. While changes in lung volumes occur in microgravity, efficient gas exchange remains and the lungs perform as they would on Earth; however, little is known about the cellular response to microgravity. In addition to spaceflight and real microgravity, devices, such as clinostats and random positioning machines, are used to simulate microgravity to study cellular responses on the ground. Differential expression of cell adhesion and extracellular matrix molecules has been found in real and simulated microgravity. Immune dysregulation is a known consequence of space travel that includes changes in immune cell morphology, function, and number, which increases susceptibility to infections. However, the majority of in vitro studies do not have a specific respiratory focus. These studies are needed to fully understand the impact of microgravity on the function of the respiratory system in different conditions.

## 1. Introduction

The lung is a uniquely dynamic organ, sensitive to endogenous and exogenous changes, including hydrostatic pressure, mechanical forces, exposure to foreign particulates, and shear stress resulting from the continuous flow of air and blood. Additionally, the lung has one of the greatest degrees of elasticity which is also influenced by the stiffness of the extracellular matrix (ECM), making the complexity of the respiratory system even greater [1]. Much of the structure of the lung is influenced by gravity, in that the lung deforms under its own weight, resulting in structural and functional differences across various regions of the lung [2]. It is then reasonable to say that the lung is highly sensitive to changes in gravitational force, not only due to the complexities of its internal structure but also due to the large surface area exposed to the external environment [2]. This review will detail what we know about how gravity, or the lack of it in space under microgravity conditions, impacts the respiratory system.

Despite being the weakest of the four fundamental forces, gravity is responsible for the formation of the universe as well as the structure of all life on Earth, including the human body [3]. Gravity is also the only constant environmental factor that was present during the evolutionary period of life and has remained unchanged over the last four billion years [4]. Due to this, there is no expected genetic memory for terrestrial life to respond to gravitational force changes. Therefore, understanding the responses of the respiratory system to microgravity will not only help in protecting the health of astronauts but also provide detailed knowledge and insight into basic cellular mechanisms and physiology with potential Earth-based benefits. 

## 2. Lung Function in Microgravity

Human physiological changes and adaptations to space conditions including microgravity have been studied in short-duration (<one month) and longer-duration (>four months) spaceflights, particularly in relation to cardiovascular and musculoskeletal health. Short- and long-duration space missions have made it clear that humans adapt to space, and microgravity is not life-threatening when the appropriate protocols are in place. Some common measurements taken during spaceflight are physical (height, body mass, heart rate, blood pressure, respiratory rate), haematological (lymphocyte, erythrocyte, and platelet count), and biochemical (electrolyte count, urine analysis, stool examination), unless a specific experiment requires additional measurements to be taken [3]. From those studies, we know that microgravity leads to cardiovascular dysfunction, muscle atrophy, loss of bone density, and immune dysfunction [5]. Some studies of lung function in microgravity have been conducted under various conditions and durations, including parabolic flights, which provide approximately 25 s of real free-fall (0 g) at the apex of the parabola, in between 20 s periods of hyper-gravity (1.8 g) during the ascent and descent of the parabola [6], and those which occurred during early space shuttle missions, at the Skylab space station, the Russian space station Mir, and the International Space Station. An important source of information about the impacts of space travel and microgravity is produced from the health monitoring of astronauts in space and on return to Earth [3].

The lung is a network of airways, alveoli, and blood vessels, which in Earth’s gravity, deforms under its own weight, so the structure of the lung is more condensed at the bottom than at the top [2]. This results in greater ventilation and perfusion at the bottom or dependent region of the lung compared to the top, where the alveoli are greatly expanded and there is very little perfusion. This heterogeneity results in heterogeneity in the ventilation–perfusion ratio throughout the lung [7]. A ‘slinky model’ has been used to demonstrate the impact of microgravity on lung structure [8]. When a slinky is held on Earth, the distance between each section is smaller at the bottom compared to the top, much like the lung as gravity pulls it down. A slinky held in microgravity on a parabolic flight showed that the distance between each section was uniform from top to bottom, as the influence of gravity was removed [8]. A similar phenomenon occurs in the lung, where more uniform alveolar expansion and blood flow occur throughout all regions, reducing heterogeneity [8]. From respiratory studies conducted during deep sea diving, it is known that the removal of gravity dependence for blood flow allows for homogenous lung perfusion [9]. This reduction in heterogeneity would lead one to assume that the heterogeneity of the ventilation–perfusion ratio would also be reduced. However, surprisingly, this has not been shown to be the case, and some heterogeneity remains in microgravity. Gravity and microgravity impose similar effects on ventilation and perfusion, so the matching between them and the efficiency of gas exchange is maintained during spaceflight [8]. This also highlights the importance of non-gravitational-dependent mechanisms in ventilation and perfusion which suggest that the sustained heterogeneity in the ventilation–perfusion ratio is not caused by the removal of gravitationally induced structural heterogeneity in the lung [10]. 

Much like how gravity is responsible for pulling objects towards the ground, it also causes blood to pool in the lower extremities of the body [10]. Therefore, the removal of gravity causes an upward shift of blood and fluids into the thorax, which has various effects on the respiratory system. Firstly, the upward shift causes an increase in pulmonary blood volume. As the lungs receive approximately 100% of the cardiac output (CO), the initial increase in CO of astronauts increases pulmonary blood flow [7,10]. Additionally, the removal of gravity allows for more uniform alveolar expansion, resulting in a greater surface area exposed to the external environment. This leads to decreased alveolar dead space and greater ventilation in the upper region of the lung compared to 1G [8]. This increase in ventilation, in addition to the 28% increase in pulmonary blood volume, causes an increase in the diffusing capacity of the alveoli by approximately 28% [2,10]. This increase remains elevated during the entire duration of microgravity exposure [2]. The removal of the hydrostatic pressure gradient within the pulmonary vasculature during microgravity exposure allows the entire pulmonary vasculature to participate in gas exchange [2]. The increase in membrane surface area for gas exchange due to increased homogeneity in alveolar expansion, the increased participation of the pulmonary vasculature, and the simultaneous increase in pulmonary blood volume further explain the increase in diffusing capacity [2].

Furthermore, the increased fluid in the thorax in microgravity takes up space that is occupied by the vital capacity in Earth’s gravity. This causes a reduction in vital capacity by approximately 10% during the initial days in space [2]. This returns to pre-flight values after an astronaut has adjusted to the fluid shift and microgravity conditions [7]. Tidal volume was found to decrease by 15% (approximately 90 mL) during the entire time of microgravity exposure [2]. Additionally, microgravity can result in breathing pattern changes for astronauts [8]. Although no studies have directly investigated chest and abdominal wall mechanics, it has been recorded that there is an increased contribution from abdominal wall muscles during tidal breathing, as the weight of the abdominal muscles and shoulder girdle is removed, altering the contributions of the inspiratory muscles [8]. However, inspiratory muscles were shown to not lose strength over periods of microgravity exposure, unlike other muscle groups. Abdominal wall compliance increases; however, rib compliance does not change, with only a slight shape change resulting in a more circular rib shape [8]. This also contributes to a respiratory rate increase of 9%. The tidal volume decrease and simultaneous respiratory rate increase result in a total ventilation decrease of 7% [8,10]. However, despite the decrease in tidal volume, alveolar ventilation remained unchanged during microgravity exposure [10].

Residual volume, measured in astronauts on Skylab 1 and 2, was reduced by 18%, approximately 310mL. This was interesting due to the typical resistance of residual volume to changes in body posture and other manoeuvres, including water immersion and G-suit inflation [10]. This change may be due to the increased uniformity in the lung resulting in uniform emptying during expiration. Additionally, the removal of gravity removes the inspiratory force produced by the weight of the abdominal muscles which contributed to a 15% (500 mL) decrease in the functional residual capacity found in the same astronauts [10]. These reductions are also predicted to be due to the upward shift of the diaphragm, increased pulmonary blood volume, and the uniformity of alveolar expansion [11]. 

The elastic recoil of the lung, responsible for the deflation and inflation of the lung, was previously determined to be a gravity-independent mechanism. Therefore, the elastic properties of the lung dominate any effects of gravity during tidal breathing. However, as gravity is removed, the distortion of lung parenchyma is also removed, resulting in a decrease in lung recoil pressure [12]. Similarly, compression of the lungs and mediastinal structures induced by gravity is lost in microgravity, causing the pressure outside of the heart and great vessels to decrease leading to the decreased central venous pressure found in astronauts in microgravity [10].

Although lung volumes have been shown to change significantly during microgravity exposure, the interpretation of results from parabolic flights has inherent limitations due to the short periods of microgravity between periods of hyper-gravity. Additionally, the Space Shuttle, Skylab, and some ISS missions only produced results for relatively short-term microgravity exposure. Despite lung function studies showing significant changes to lung volumes and perfusion, the lungs perform efficient gas exchange and function as they would in 1G on Earth, indicating that microgravity-induced changes to lung function are due to anatomical adaptations rather than functional changes.

## 3. Methodologies for Studying Microgravity on Earth

**Figure 1:**
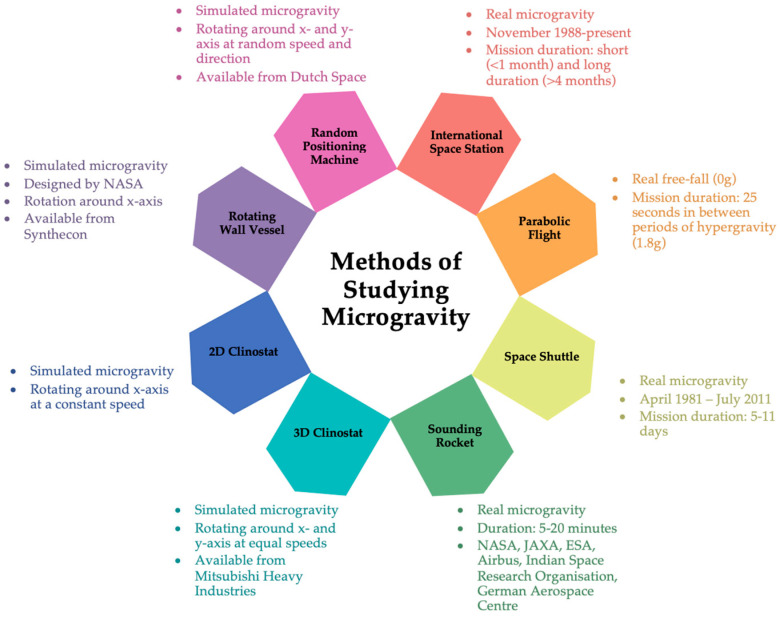
A summary of the methods of studying microgravity on Earth and in space.

To obtain an idea of how microgravity might change cellular functions, ground-based microgravity simulators like those presented in Figure 1, can be used, with the understanding that the results need final validations in real microgravity. Clinorotation was the first and is the most common method of simulating microgravity. The principle of clinorotation is based on the redistribution of the gravity vector in a circle. The first clinostat was developed by Julius Von Sachs in 1879, who produced a clockwork-powered machine to study the effects of gravity on the growth of plants [13]. This first device has been modified and developed over time as technology has improved and multiple devices are in use today. Clinorotation generates centrifugal forces that vary depending on rotational speed and the distance of the sample from the rotation axis [14]. A two-dimensional (2D) clinostat functions by rotating cells continuously around the horizontal axis, causing the gravity vector to continuously change with respect to the direction of the cells, such that the cells constantly change their direction. The slow rotating (1–4 rpm) clinostat is typically used to study plant growth and development as the response time of plants is relatively slow. The gravitropic responses are suppressed at slow rotation. However, it is suggested that slow rotation may create a stressful environment as the circular radius of the statolith may be equal to or greater than the size of the cell itself, thus generating pressure on the cell wall [15]. Fast-rotating (50–120 rpm) clinostats are used for small particles within a fluid. The slow rotation of particles in a fluid results in unwanted stresses. When the speed of rotation is increased but still constant, sedimentation will be slower than fluid movement; thus, sedimentation will be prevented and the particle will rotate around its own centre with stabilised fluid movement around the particle [15,16]. For the best results, rotational speed should result in sedimentation and centrifugal force to fall within Brownian motion [16]. Therefore, with appropriate rotational speed and the minimisation of centrifugal forces, the cells always experience a delayed response to gravity, thereby simulating a microgravity environment of 10^−3^ g [14,17].

A rotating-wall vessel (RWV) or rotary cell culture system (RCCS) share similarities with the 2D clinostat in that there is a horizontally rotating cylindrical culture vessel but with a coaxial tubular oxygenator. Rotating around the horizontal axis at a defined rotational speed regulates the fall velocity to keep the sample in a constant free-fall to simulate microgravity between 10^−2^ and 10^−3^ g [14,17]. Under these conditions, cells can aggregate and begin to form tissue-like spheroids [18]. The fluid flow in a rotating-wall vessel is laminar in most operating conditions which avoids large shear stresses associated with turbulent flow. As the vessel rotates, the fluid inside accelerates until the entire fluid mass is rotating at the same angular rate as the wall [19]. As the culture medium is mixed gently through rotation, the need for stirring vanes is eliminated [19]. Like the 2D clinostat, the rotating-wall vessel needs to be completely filled with culture medium, as incomplete filling leads to air in the vessel that creates turbulence and bubble formation that are large sources of extra shear stress [19].

The rotating-wall vessel relies on solid body rotation about a horizontal axis, resulting in the colocalisation of cells and aggregates of different sedimentation rates and reduced shear and turbulence [19]. NASA first designed the rotary cell culture system to protect cell culture experiments from the high shear forces generated during the launch and landing of the space shuttle missions to ensure reliability and accuracy in experiments [18,19]. The NASA-engineered rotating-wall vessel was produced with low shear and laminar flow conditions to develop a robust model system of the conditions predicted for cell culture in space.

The 3D clinostat and random positioning machine (RPM) apply the same principles as the 2D clinostat with the addition of another axis of rotation. In addition to rotating around the horizontal axis, the 3D clinostat and RPM have a second frame that independently rotates around the vertical axis. The 3D clinostat rotates at a constant speed and direction while the RPM rotates at a random speed and direction. For the RPM, randomness is achieved when the rotational angle differs between the two axes and changes over time, thus distributing the gravity vector in all directions over time to simulate microgravity between 10^−4^ and 10^−5^ g, depending on the settings and device [14,15,17]. A consideration to be made when using these devices is the vibrations generated that may impact the results. However, all these devices are suitable for exposing cultured mammalian cells to microgravity with further validation under real microgravity conditions [16].

While these devices allow ground-based research to be conducted investigating the effects of microgravity in vitro, they do not come without limitations. For all the devices, the full vessel environment leads to changes in the hydromechanical environment and affects oxygen delivery to cells with downstream cell signalling impacts [20]. During standard static experiments, cells will experience atmospheric pressure and hydrostatic pressure from the culture medium. However, the fluid motion during simulated microgravity results in convection and flow-induced shear stress [20]. Therefore, the flow dynamics of each microgravity simulator device should be known before experiments are conducted and the impact of forces from fluid motion on cellular mechanoreceptors should be considered when explaining the results.

The air–liquid interface of the culture medium during standard cell culture experiments is responsible for oxygen exchange and balancing of culture medium pH. During full-vessel experiments, static or microgravity, the surface area available for oxygen exchange is reduced. However, the convective mixing during microgravity exposure improves mass transport and oxygen dissolution [20]. Therefore, some effects of simulated microgravity on cell function may not be present when validating the results in real microgravity.

The cell type should also be considered for each microgravity simulator device. For example, adherent cells on the rpm will experience shear forces on the vessel wall that may be responsible for the results obtained rather than the impact of microgravity [20]. Adherent cells are also likely to detach from the culture vessel, which introduces additional considerations of whether exposing detached cells to microgravity is a relevant model. Whether the results from adherent cells are due to the shear stress of simulated microgravity or the reduced ability to adhere to substrates under microgravity conditions needs to be elucidated.

With the effects of microgravity simulator devices to be considered, having multiple controls can identify the non-gravitational effects of these devices, including shear forces, vibrations, fluid flow, and oxygen delivery. The controls should be meticulously considered, and some can include standard static, full vessel static, standard dynamic, and full vessel dynamic. The benefits and limitations of each device should be carefully considered for specific experiments, and all non-gravitational effects should be accounted for in the interpretation of data.

## 4. Microgravity Impacts Cellular Communication with the Microenvironment

Cells contain various organelles and components which all have a mass and therefore, are influenced by gravity [21]. Microgravity can affect cellular behaviour directly through response elements within the cell or by influencing and changing the surrounding environment of the cells. Cogoli and Cogoli-Greuter developed three theoretical models of cells sensing gravitational changes [4]. The first is a direct effect where gravity interacts with cellular organelles which have a different density to the cytoplasm, thus generating pressure on the surrounding structures, resulting in signal transduction and biological effects. Under microgravity conditions, it is unknown how that changes cellular function. An indirect effect involves gravity- or microgravity-induced changes to the microenvironment of the cells and the cell response to the new conditions. The final theory is a non-equilibrium thermodynamic effect where gravity interacting with a small number of organelles is insufficient to cause an adverse biological effect, but a series of small changes are amplified to produce a greater effect with functional changes [4,22].

Further investigations have highlighted that in human cells, the ECM and the cytoskeleton form the foundation for gravitational sensing [23]. The cytoskeleton communicates with the extracellular matrix through focal adhesions and thus can detect and initiate responses to mechanical changes in the cellular environment, such as changes in gravitational force [23]. However, the mechanisms of how these mechanical signals are transformed into biochemical signals under changing gravity conditions remain an unanswered question. 

The tensegrity model developed by Ingber describes how cells might sense microgravity [24]. Under gravity conditions, the ECM and cells communicate through focal adhesions and the cytoskeleton. However, under microgravity conditions, this communication is disrupted as the balance between those forces is altered. This can lead to morphological changes as well as activation of regulatory proteins such as integrins, resulting in changes in cell signalling cascades and gene expression [23,24]. While no gravireceptor or specific mechanism for sensing and responding to changes in gravitational force has been identified and this research is ongoing, the tensegrity model suggests that instead of activating a single gravireceptor, changes to cytoskeletal elements lead to stress-related morphological changes [24]. However, whether cell-morphology-change-induced changes in intracellular biochemical signals can ultimately influence the communication between cells and their microenvironment remains unknown. However, studies have been conducted highlighting the importance of the ECM in maintaining homeostasis throughout the body, specifically within the lung. How this is altered under microgravity conditions will be discussed below.

Research suggests that osteocytes, chondrocytes, and fibroblasts, important ECM-producing cells, are mechanosensitive and respond to gravitational changes [25]. Tissues, including the lungs, are remodelled through interactions between cells and their microenvironment. Throughout the body as well as the lung, stable adhesion during cell–cell and cell–ECM interactions is via adhesion molecules as well as the secretion and adsorption of soluble factors [26]. Stable adhesion maintains cell communication, epithelial integrity, and ECM integrity. Cell adhesion molecules (CAM) such as integrins, cadherins, and selectins, and other ECM proteins are involved in regulating differentiation, migration, proliferation, and survival [27]. Under microgravity conditions, these proteins can act as mechano-sensors, taking external mechanical signals and transducing them into biochemical responses, initiating downstream signalling cascades that affect the morphology and function of cells.

After spaceflight, genes associated with CAM and ECM proteins were differentially expressed [28]. An upregulation of collagen genes and downregulation of matrix metalloproteinase (MMP) genes, which are responsible for the degradation of collagen, was found after mice were flown on space shuttle missions [29]. It has been shown that after real microgravity conditions, fibroblasts increase collagen synthesis [30]. In theory, within the lung, this would lead to increased collagen deposition, increasing the stiffness of the ECM and the lung. Increased collagen deposition and ECM stiffness are hallmark characteristics of pulmonary fibrosis.

Fibroblast–epithelial interactions are essential for the maintenance of homeostasis, inflammation, proliferation, and tissue remodelling through mechanotransduction of stimuli and modification of ECM components [31]. In the context of the lungs, fibroblast–epithelial interactions are critical for wound healing and maintaining healthy ECM deposition and composition [32]. While there are few studies directly investigating lung fibroblasts, studies have reported that RPM-simulated microgravity impacts the shape and orientation of fibroblasts which disrupts cell alignments and alters their adhesion to substrates [31]. Dermal fibroblasts, after 24 h of RPM-simulated microgravity exposure, have decreased proliferation and increased apoptosis. Additionally, there was a decrease in the S phase cell cycle and an increase in the G2 and M phases [31]. This aligns with gene expression data from lung fibroblasts flown on the space shuttle, where there was an upregulation of pro-apoptotic gene expression as shown in Table 1. This aligns with another study which found a downregulation of cell cycle and proliferation genes including *p21* and *ERB-B2* after 3D clinostat microgravity exposure [30]. Furthermore, it was found that RPM-simulated microgravity impairs the migratory ability of fibroblasts after a significant decrease in migration and invasion was reported. However, this returned to normal after 72 h of microgravity exposure, suggesting that migratory properties are adaptive [31]. 

It was found that under in vitro co-culture conditions, dermal fibroblast–keratinocyte interactions were disrupted, and the cells were partitioned into distinct layers during simulated microgravity exposure on the RPM [31]. It is reasonable to speculate that a similar disruption may occur in the lungs between lung fibroblasts and epithelial cells. Impaired mechanotransduction can interfere with cross talk between many cell types and the ECM which will influence cell behaviour and lung function [31]. From studies of lung diseases, it is known that alterations in ECM deposition and composition are found in idiopathic pulmonary fibrosis, chronic obstructive pulmonary disease, and asthma [26,32,36]. Therefore, microgravity might change normal lung tissue organisation which alters the physiological function of the respiratory system.

## 5. Immune Function under Microgravity Conditions

An emerging field of research is the lung microbiome, which unlike the gut microbiome is a dynamic relationship. The upper respiratory tract is largely colonised by bacteria, and even within the upper respiratory tract, there are topographical differences [37]. Conversely, the lower respiratory tract has a low biomass largely due to rapid microbial clearance allowing the lower respiratory tract to maintain effective oxygen and carbon dioxide exchange, a crucial function for sustaining life [37]. Lung microbiome dysbiosis has been linked to the development and progression of respiratory diseases such as COPD, asthma, cystic fibrosis, and IPF. The respiratory system relies heavily on clearance mechanisms including mucociliary clearance, cough, and innate and adaptive immune responses to maintain a low bacterial burden [37]. Microorganisms have been found to thrive in spaceflight conditions including microgravity, in terms of sustained or increased proliferation even with normally inhibitory amounts of antibiotics [38]. Microgravity has the potential to make some microbes more susceptible to cause disease. While it is currently unknown if the effectiveness of mucociliary clearance and cough is maintained in microgravity conditions, microbes are more likely to remain aerosolised in microgravity conditions leading to an increased risk of spreading among crewmates. Innate and adaptive immune system function is critical for both systemic and respiratory health. This section will detail some of the changes found in these systems during microgravity exposure.

A well-studied impact of microgravity exposure is disruptions to the immune system; however, the direct consequences of this disruption to the response to infection in a systemic and respiratory context are less well understood. After Apollo 7, astronaut Wally Schirra developed a cold during the mission, which was later passed onto the other astronauts; NASA developed a week-long quarantine requirement before missions to protect astronauts from pathogens that could lead to infections [39]. Due to the confined nature of space travel, infections spread rapidly between crewmates which poses a significant threat to the success of the mission. Despite this quarantine protocol, respiratory infections have been reported during spaceflight. Respiratory infections, microgravity-induced upward fluid shift, and dry cabin atmosphere share many of the same symptoms making the diagnosis of infection difficult. The upper respiratory symptoms include rhinitis, prolonged congestion, and sneezing, which were some of the most common immune-related health events experienced during spaceflight [40].

It has been suggested that microgravity can directly affect viral stability and survival or indirectly impact the host’s vulnerability and response to infection. Additionally, factors including temperature and pressure in space, especially on lunar missions, can influence viral activity and pathogenic effect. During shuttle missions (10–16 days) and ISS missions (>180 days), latent virus reactivation has occurred in the majority of astronauts. After reactivation has occurred, viral DNA is shed in body fluids [41]. While it is typical for astronauts to be asymptomatic following viral shedding, live and infectious viruses have been detected in tissues from skin lesions and atopic dermatitis of astronauts [41]. Furthermore, 53% of space shuttle astronauts and 61% of ISS astronauts shed at least one herpes virus in their saliva or urine, while Epstein–Barr virus (EBV), Varicella–Zoster virus (VZV), and Herpes Simplex 1 virus have been detected in saliva and cytomegalovirus (CMV) has been detected in urine [41]. It has also been shown that viral shedding does not decrease during long-duration missions as the astronauts and the immune system adapt to space, but rather increases. From short-duration space shuttle missions to long-duration ISS missions, viral shedding increased from 41 to 65% for VZV, 82 to 96% for EBV, and 47 to 61% for CMV [41]. These viruses as well as other pathogens can be aerosolised and remain aerosolised for long periods of time under microgravity conditions. While the reactivation of these viruses has typically remained asymptomatic, some astronauts develop skin lesions and there have been six reported incidences of shingles resulting from the reactivation of herpes virus. CMV is also suggested to be immunosuppressive which may be a crucial part of the immune dysfunction experienced in microgravity [41]. The pre-flight quarantine period is not a preventative measure against the reactivation of latent viruses. Viral reactivation is associated with the stress that arises from microgravity as well as other spaceflight stressors including isolation and confinement, anxiety, sleep deprivation, physical exertion, and radiation. Viral reactivation increases the risk of adverse medical events occurring during spaceflight and deep-space missions [42]. While these are not directly respiratory viruses, the lung contains many viruses and other pathogens which could be reactivated or may lead to the development of symptoms.

From the analysis of whole blood and immune cells of astronauts, more specific information about immune cell changes due to microgravity exposure is known. Following real and simulated (RCCS) microgravity exposure, there is a decreased number of macrophages, and they exhibit impaired polarisation. These macrophages also have decreased oxidative burst, metabolic alterations, and cytoskeletal changes [43]. In the context of the respiratory system, macrophages are responsible for the phagocytosis of foreign particles, pathogens, and dead cells, which is an extremely important process to the resolution of inflammatory responses. Therefore, dysfunction of these cells can lead to increased susceptibility to infection as well as sustained inflammation [44]. Similarly, dendritic cells isolated from astronauts on a Soyuz mission in addition to dendritic cells cultured on RCCS showed microgravity-induced changes. Although there have been conflicting results regarding the number of dendritic cells, studies align with dendritic cells exhibiting a decreased phagocytic and antigen presentation ability which have important impacts on the clearance of foreign particles or pathogens and the initiation of immune responses within the lungs [43]. Another important immune cell type is neutrophils which operate as a link between the innate and adaptive immune systems and play an important role in respiratory diseases. Following real and simulated microgravity exposure, neutrophils displayed morphological changes in addition to increased numbers. Like dendritic cells, the phagocytic ability of neutrophils decreased, further highlighting that microgravity induces functional changes in the immune system [43].

Within the adaptive immune system, B and T cells are crucial components. From the limited studies available, there are conflicting results regarding B cell numbers. Mice flown for one month in space had a decreased number of B cells, while astronauts aboard the ISS showed no change in B cell numbers and homeostasis was maintained [43,45,46]. Furthermore, samples from various space shuttle astronauts showed a decreased number of T cells, and the cells had decreased cytokine secretion, including IFN-gamma, IL-1beta, IL-4, IL-10, and IL-12 [47]. However, the production of IL-8 and TNF-alpha was greater following mitogenic stimulation compared to the 1G Earth control. Functional responses to bacterial and viral infection were reduced during and following microgravity exposure. Microgravity also causes impaired T cell activation by impacting the ability of cells like neutrophils and macrophages to activate T cells, but also by altering the T cells [47].

The number of monocytes from astronauts on space shuttle missions did not change; however, they did have decreased phagocytic ability, specifically in engulfing Escherichia coli. Additionally, the monocytes had decreased oxidative burst and degranulation capacity, thus highlighting the impaired function induced by microgravity [43,48]. The monocytes additionally exhibited decreased responses to Gram-negative endotoxins, specifically a decreased production of cytokines IL-8, IL-6, IL-1ra, and IL-1beta. Toll-like receptor 4 and CD14 expression levels were also decreased in astronaut monocytes compared to non-astronaut monocytes [49]. 

Natural killer (NK) cells cultured under simulated microgravity conditions using RWV had a decreased cytotoxicity accompanied by increased apoptosis and necrosis [50]. NK cells from astronauts aboard a long-duration (6 months) mission had decreased killing efficiency, with newer astronauts having greater impairment compared to both control and veteran astronauts [51]. NK cells are potent effectors of the innate immune system and are important for the surveillance of latent viral infections and tumours [51]. Thus, impairment of these cells negatively impacts immune function in space.

## 6. Conclusions

Despite some changes in lung volumes, the lungs maintain efficient gas exchange in microgravity conditions. However, previous studies do not go beyond lung function tests, and in vitro experiments are limited to immune cell and cell adhesion experiments without a specific respiratory focus. The current knowledge regarding the functional changes of the lungs and residing cells of the respiratory system in microgravity conditions is lacking. Further research is required to elucidate how microgravity impacts cellular communication between cells and with the microenvironment and downstream signalling effects. Overall, dysregulation of the immune system and dysbiosis of the lung microbiome under microgravity conditions may have greater implications for respiratory function; however, further research is required to identify the mechanisms. Additionally, other space stressors such as radiation, physical, and psychological stress should be studied in combination with microgravity to provide a complete understanding of how microgravity impacts the respiratory system.

## Figures and Tables

**Table 1:** A summary of the microgravity-induced effects on lung-specific cell types.

Cell Type	Microgravity Exposure	Exposure Duration	Cellular Effects	Reference
Bronchial epithelial cell (BEAS-2B)	3D clinostat	48 h	Reduced cell survival Reduced cell proliferation Increased apoptosis Increased reactive oxygen species	[33]
Alveolar epithelial cell (A549)	RPM	24–48 h	Increased polynucleated cells Altered mitochondria morphology	[34]
Lung fibroblast (WI-38)	STS-93 (space shuttle)	4 days 23 h	Upregulated expression of genes associated with stress response signalling, cell-cycle re-entry, and pro-apoptosis signalling Downregulation of genes associated with energy metabolism	[35]
